# Impact of Russian current therapy targeting the anterior tibial group on balance and fall risk in post-stroke patients: a randomised controlled trial

**DOI:** 10.1007/s10072-026-08959-9

**Published:** 2026-04-23

**Authors:** Ashraf A. Darwesh, Abdelrahman Refaat Mohamed, Hoda Mohamed Zakaria, Ebtesam Mohamed Fahmy, Zizi M. Ibrahim, Olfat Ibrahim Ali, Mohamed Abdelaziz Emam, Doaa Youssry, Mahmoud Yassin Elzanaty

**Affiliations:** 1 Department of Physical Therapy for Neurology, College of Physical Therapy, Cairo University, Cairo, 12613 Egypt; 2 Department of Physical Therapy for Neuromuscular Disorders and Its Surgery, Faculty of Physical Therapy, Deraya University, Minya, Egypt; 3 Professor of Neurology, Faculty of Medicine, Cairo University, Kasr Al-Aini, Cairo, Egypt; 4 Department of Rehabilitation Sciences, College of Health and Rehabilitation Sciences, Princess Nourah bint Abdulrahman University, P.O. Box 84428, Riyadh, 11671 Saudi Arabia; 5 Physical Therapy Program, Batterjee Medical College, Jeddah, 21442 Saudi Arabia; 6 Basic Sciences Department, Faculty of Physical Therapy, Kafrelsheikh University, Kafrelsheikh, 33511 Egypt; 7 János Szentágothai Neurosciences Division, Semmelweis University, Budapest, 1085 Hungary; 8 Faculty of Medicine, Cairo University, Cairo, Egypt; 9 Physical Therapy for Neuromuscular disorders and its surgery, Faculty of Physical Therapy, Cairo University, Cairo , Egypt; 10 Physical Therapy for Neuromuscular disorders and its surgery, Faculty of Physical Therapy, Deraya University, Minya, Egypt

**Keywords:** Anterior tibial group, Berg balance scale, Biodex-balance system, Overall stability, Postural stability, Risk of fall, Russian current, Stroke

## Abstract

**Background:**

Post-stroke patients often suffer from impaired postural stability and a heightened risk of falls due to neu- romuscular deficits. Russian current stimulation, a medium-frequency neuromuscular electrical stimulation technique, has shown promise in enhancing muscle strength and mo- tor control.

**Purpose:**

This study aimed to investigate the effect of Russian current stimulation applied to the ante-rior tibial muscle group, in conjunction with a structured physiotherapy program, on postural stability and fall risk in stroke survivors.

**Methods:**

A randomized controlled trial was conducted with 40 stroke patients aged 40–60 years (BMI < 30 kg/m²), divided equally into study (n=20) and control (n=20) groups. Both groups received a standard 40-minute physiotherapy regimen three times weekly for six weeks. The study group also received Russian current stimulation (2.5 kHz, 50 bursts/sec, 20 min/session) target- ing the anterior tibial group. Outcomes were assessed pre- and post-intervention using the Biodex Balance System in- dices (overall, anterior-posterior, and medial-lateral stabil- ity), and fall risk index

**Results:**

Significant improvements were observed in the study group compared to controls as reductions in overall stability index, anterior-posterior and medial-lateral sway indices (all p < 0.05). No significant dif- ference was noted between groups in fall risk under eyes- closed conditions (p > 0.05).

**Conclusion:**

The addition of Russian current stimulation to conventional physical therapy significantly improves postural stability and reduces fall risk in stroke patients under eyes-open conditions. These findings support the integration of neuromuscular electrical stimulation into stroke rehabilitation protocols targeting the anterior tibial muscle group.

**Trial registration:**

Clinical-Trials.gov Identifier: NCT06793865

## Introduction

Stroke is still one of the major global causes of adult disability, and it remains the second leading cause of death in the world, with an approximate share of 11.13% of total deaths [1]. Egypt has a crude prevalence rate of stroke that reaches 963 per 100,000 people, which means that stroke has become a significant public health issue in the country.[2]. Among numerous post-stroke complications, impaired postural control and a higher tendency to fall are certainly among the most common and most debilitating sequelae. For instance, within one week after suffering from a stroke, about 7% of survivors will experience falls, increasing to around 73% within one year after discharge from hospital [3]. The occurrence of falls severely affects an individual’s safety and sense of independence. Additionally, these individuals tend to be hospitalized more often, where they are required to undergo lengthy rehabilitation processes, resulting in a diminished quality of life.

Post-stroke impairments—such as muscle weakness, sensory deficits, and diminished motor coordination—contribute to balance dysfunction and gait abnormalities. These challenges are often exacerbated by asymmetrical body alignment and abnormal postural reflexes, leading to decreased mobility and greater dependency in activities of daily living (ADLs) [4, 5]. Furthermore, high fall rates are commonly associated with impaired balance, which places a heavy burden on stroke patients, their families, and society as a whole. Restoring balance is important in effectively performing ADLs and ambulating independently [6].

A critical component of post-stroke gait recovery is dorsiflexion during the swing phase of walking, which is pri- marily governed by the tibialis anterior (TA) muscle. The common “foot drop” condition that occurs post-stroke due to weakness or paralysis of this muscle increases postural instability and greatly impairs one’s ability to walk [7]. Tradi- tional physiotherapy, including muscle strengthening and balance exercises, has shown benefits; however, the extent to which these interventions restore motor control and prevent falls remains limited [8]. Neuromuscular electrical stimulation (NMES), particularly Russian current (RC) stimulation, a medium-frequency alternating current (2.5 kHz)—has been increasingly adopted in neurorehabilitation to enhance muscle activation, promote motor recovery, and prevent disuse atrophy. Russian current is well tolerated, less painful, and known for its ability to recruit a larger number of motor units simultaneously, thereby facilitating greater muscle strength gains compared to conventional methods [8, 9].

While its efficacy has been demonstrated in orthopedic and athletic populations, its role in post-stroke rehabilitation, especially for improving static and dynamic balance, requires further investigation. Given the high incidence of postural instability and fall-related injuries among stroke survivors, and the growing interest in NMES modalities, this study aimed to evaluate the impact that Russian current stimulation applied to the anterior tibial muscle group would have on postural stability and fall risk in post-stroke patients. Balance outcomes in this study were assessed using the Biodex Balance System, a computerized dynamic posturography tool that objectively quantifies postural stability through indices such as the Overall Stability Index, Anterior-Posterior Stability Index, and Medial-Lateral Stability Index under various sensory conditions. It also provides a Fall Risk Index, offering a comprehensive evaluation of balance impairments [10]. This study investigates the impact of adding Russian current stimulation to a conventional physiotherapy program in stroke rehabilitation. By comparing this combined approach to physiotherapy alone, the research aims to inform evidence-based strategies for enhancing neuromotor function and minimizing fall risk in stroke rehabilitation. It is hypothesized that the integration of Russian current stimulation with conventional therapy will lead to significantly greater improvements in postural stability and a reduction in fall risk among post-stroke patients compared to conventional physiotherapy alone.

## Methods

### Study design

This study was a single, blinded-randomized controlled trial (assessor was blinded). Conducted in the Outpatient Clinic of Neurological Physical Therapy Department in Deraya University, Egypt. Starting from October 2024 to November 2025.

#### Subject

Forty patients of both genders, diagnosed with stroke, were included according to the following criteria, they were aged from 40 to 60 years old; their body mass index (BMI) did not exceed 30 kg/m2, their affection distal is more than proximal with moderate spasticity, they complained of weakness of the pretibial group, they were medically and clinically stable. Patients were excluded if they were suffering from psychological, cognitive, or emotional disturbance, severe spasticity (3 based om modified Ashworth scale), atrial fibrillation, having an infection, abnormal pain sensitivity, epilepsy, or lower extremity articulations with endoprothetics that may interfere with subject treatment.

### Randomization

Each participant was informed about the nature and goals, and they were also given the option to stop participating at any time. Demographic data were collected once consent forms were signed. Next, an additional researcher used opaque envelopes with computer-generated random numbers on them to divide the 40 subjects evenly between the two groups: the study group and the control group. The envelopes were sealed and sequentially numbered to ensure the concealed allocation, and participants were unaware of their group allocation.

#### Study group

20 stroke patients received RC(fifty bursts every second, with fifty pulses in each burst, with 2.5Khz frequency; applied for 10 s followed by the same period, as a rest along 20 min, and a selected physical therapy program involved passive, active assisted, in addition to active range of motion ROM, stretching, and strengthening exercises for the spastic ‘anterior tibial group’ plus functional mobility exercises along 40 min.; 3 sessions per week for 6 weeks.

Control group: 20 stroke patients were given a placebo RC for 20 min, and the same selected physical therapy program was given to the study group, 3 sessions per week for 6 weeks. Sample size calculation: Twenty participants per group were determined to be the sample size for this study by utilizing G*POWER statistical software (version 3.1.9.2; Franz Faul, Universität Kiel, Germany) in conjunction with the overall stability index data obtained from Elsodany et al. [11], who investigated a similar intervention of electrical stimulation on the anterior tibial group. A power of 80% and an effect size of 0.91 are used in the calculation, with α = 0.05.

#### Control group

20 stroke patients were given a placebo RC for 20 minutes, and the same selected physical therapy program was given to the study group, 3 sessions per week for 6 weeks. Sample size calculation: Twenty participants per group were determined to be the sample size for this study by utilizing G*POWER statistical software (version 3.1.9.2; Franz Faul, Universität Kiel, Germany) in conjunction with the overall stability index data obtained from Elsodany et al. [11], who investigated a similar intervention of electrical stimulation on the anterior tibial group. A power of 80% and an effect size of 0.91 are used in the calculation, with α =0.05.

#### Ethical Statement

Clinical trial registration number (NCT06793865) as well as the Local Ethical Committee of the Faculty of Physical Therapy at Cairo University (No: P.T.REC/012/005504) both gave their approval to the study.

### Instrumentations

Biodex-balance system (TP-3040-15, DC input: 19V-6. 32A software version 4.0.06, 2017/ SR-R712010010; Taiwan) is a valid and reliable device [12] used as an objective assessment of both postural stability and measure risk of fall (ROF) indexes[13]. Measurements were carried out before and after the 6-week intervention.

Es-5200 double-channel electrotherapy device (ITO Co., Ltd, 2017) was utilized for RC stimulation in the study group, and a placebo RC application in the control group.

### Procedures

#### Assessment procedures

 Postural stability assessment using the Biodex-balance system in terms of overall stability index (OSI), anterior-Posterior Stability Index (APSI), and medial-Lateral Stability Index (MLSI); Every patient was administered a training test before being subjected to 3 successive static mode tests. The devices automatically computed the mean of three tests demonstrating the postural variations of women; each test extended 20 s with only a 10-second rest in between. Variations were recorded with respect to the platform’s center.

Risk of falls assessment: All patients in both groups were asked to bilaterally stand on the locked platform; Par- ticipants in both the study and control groups completed the following tasks at baseline and post the 6-week intervention: looking at a height-adjustable screen, placing their hands comfortably by their sides, and being instructed to utilize the device’s handles in the event of a loss of balance.

#### Therapeutic procedures

All participants were informed about the purpose and procedures of the selected physical therapy program, then received Passive, active-assisted, in addition to active ROM, stretching of spastic calf musculatures, strengthening of Tibialis anterior for the study group, and functional mobility exercises. RC session: Only Study group participants received RC for 20 minutes based on the study protocol, those were adjusted as the following frequency of 2.5 kHz and 50 bursts per second with 50 pulses per burst. The stimulus was applied for seconds, with a 10-second "on" period followed by a 10-second "off" period, for a total treatment time. The intensity of the stimulation ranged from 22 to 24 mA. Where, the control group a placebo. RC stimulation was applied to the anterior tibial group without active current for 20 minutes.

Selected physical therapy exercises for both limbs: These were applied for 40 minutes, including passive, active assisted in addition to active range of motion exercises, stretching of spastic muscles, strengthening of weak muscles (TA group), and finally functional mobility exercises.

## Results

Subject characteristics: The subjects in both groups are shown in Table 1. No statistically significant differences were found between the groups with respect to age, BMI, illness duration, genders, and distribution of affected sides (*p* > 0.05).

**Table 1:** Comparison of subject characteristics between groups

Characteristic	Study group (Mean ± SD)	Control group (Mean ± SD)	p-value
Age (years)	49.90 ± 2.73	50.40 ± 3.42	0.61
BMI (kg/m²)	26.15 ± 2.38	26.73 ± 2.09	0.42
Duration (months)	8.25 ± 1.52	8.70 ± 1.81	0.39
Sex, n (%)			
Female	10 (50%)	8 (40%)	0.53
Male	10 (50%)	12 (60%)	
Affected side, n (%)			
Right side	7 (35%)	8 (40%)	0.74
Left side	13 (65%)	12 (60%)	

*SD* Standard deviation, *m* months, p-value: Level of significance

### Effect of treatment on postural stability and fall risk

Time and treatment interacted significantly (F = 31.75, p = 0.001, Partial Eta Squared = 0.91), according to a two-way mixed MANOVA (F=187.44, p = 0.001, Partial Eta Squared = 0.98), indicating that time is a significant main effect. Treatment had a statistically significant main impact (F = 6.42, p = 0.001, Partial Eta Squared = 0.66).

### Within group comparison

The OSI, APSI, and MLSI levels of both groups significantly decreased (*p* < 0.001) with groups according to Table 2.

**Table 2:** Mean differences OSI, APSI, and MLSI values

Variable	Group	Pre (Mean ± SD)	Post (Mean ± SD)	MD (95% CI)	% Change	p-value
OSI	Study Group	4.11 ± 1.10	2.95 ± 0.73	1.16(0.82, 1.50)	28.22	0.001
	Control Group	4.15 ± 0.83	3.60 ± 0.80	0.55(0.25, 0.85)	13.25	0.001
	MD(95%CI) / p-value	-0.04(-0.669, 0.579)	-0.65(-1.141, -0.159)			0.88 / 0.01
APSI	Study Group	2.74 ± 1.13	1.87 ± 1.00	0.87(0.48, 1.26)	31.75	0.001
	Control Group	2.88 ± 0.83	2.56 ± 0.80	0.32(0.02, 0.62	11.11	0.001
	MD(95%CI) / p-value	-0.14(-0.77, 0.49)	-0.69(-1.27, -0.11)			0.66 / 0.02
MLSI	Study Group	2.39 ± 0.68	1.55 ± 0.47	0.84(0.63, 1.05	35.15	0.001
	Control Group	2.58 ± 0.56	2.19 ± 0.50	0.39(0.19, 0.59)	15.12	0.001
	MD(95%CI) / p-value	-0.19(-0.59, 0.21)	-0.64(-0.96, -0.34)			0.34 / 0.001

*MD *Mean difference,* OSI* Overall Stability Index, *APSI* Anterior-Posterior Stability Index, *MLSI* Medial-Lateral Stability Index

After treatment, the study and control groups had a significantly lower overall, AP, and ML sway index, as well as eye-opened narrow surface (EONS) velocity compared to baseline values (*p* < 0.001), but neither group showed a significant difference within eye-closed narrow surface (ECNS) velocity (*p* > 0.05). (Tables 3 and 4).

**Table 3:** Mean overall, AP, and ML sway index

Variable	Group	Pre (Mean ± SD)	Post (Mean ± SD)	MD(95%CI)	% Change	p-value
Overall sway index	Study Group	3.69 ± 0.80	2.78 ± 0.59	0.91(0.76, 1.06)	24.66	0.001
	Control Group	3.73 ± 0.95	3.32 ± 0.80	0.41(0.26, 0.56)	11.00	0.001
	MD(95%CI) / p-value	-0.04(-0.59, 0.53)	-0.54(-0.98, -0.09)			0.90 / 0.02
AP sway index	Study Group	2.91 ± 0.82	2.18 ± 0.74	0.73(0.64, 0.82)	25.09	0.001
	Control Group	2.94 ± 0.65	2.65 ± 0.64	0.20, 0.38	9.86	0.001
	MD(95%CI) / p-value	-0.03(-0.50, 0.44)	-0.47([-0.92, -0.03)			0.89 / 0.03
ML sway index	Study Group	2.35 ± 0.52	1.64 ± 0.38	0.71(0.58, 0.84)	30.21	0.001
	Control Group	2.40 ± 0.54	2.03 ± 0.45	0.37(0.24, 0.50)	15.42	0.001
	MD(95%CI) / p-value	-0.05(-0.38, 0.29)	-0.39(-0.66, -0.13)			0.77 / 0.005

*AP* Anterior-posterior, *ML* Medial-lateral

**Table 4:** Mean fall risk pre and post treatment of study and control groups

Variable	Group	Pre (Mean ± SD)	Post (Mean ± SD)	MD(95%)	% Change	p-value
EONS (mm/sec)	Study Group	15.02 ± 3.02	10.61 ± 3.17	4.41(3.79, 5.03)	29.36	0.001
	Control Group	15.33 ± 2.66	12.83 ± 2.28	2.50(1.88, 3.12)	16.31	0.001
	MD(95%CI) / p-value	-0.31(-2.13, 1.51)	-2.22(-3.98, -0.45)			0.73 / 0.01
ECNS (mm/sec)	Study Group	17.94 ± 2.64	17.79 ± 2.57	0.15(-0.07, 0.37)	0.84	0.17
	Control Group	18.79 ± 1.89	18.62 ± 1.89	0.17(-0.05, 0.39)	0.90	0.14

*SD* Standard deviation, *MD* Mean difference, *EONS* Eyes open narrow surface, *ECNS* Eyes Closed narrow surface, *p-value* Probability value

### Between group comparison

Groups did not differ significantly before treatment (*p* > 0.05). After the treatment, study group had a significantly higher BBS and significantly lower OSI, APSI, and MLSI metrics compared to control group (*p* < 0.05). According to Table 2.

After the therapy, study group had a significantly lower overall, AP, and ML sway index, and eye opened narrow surface (EONS) velocity compared to control group (*p* < 0.05). After therapy, ECNS velocities did not differ significantly (*p* > 0.05) across groups. (Tables 3 and 4).

## Discussion

This study was conducted to explore the effect of Russian current (RC) stimulation applied to the anterior tibial muscle group on postural stability and risk of falling in stroke patients, and the results revealed that postural stability was sig- nificantly improved and decreased sway indices (OSI, APSI, MLSI) in the experimental group compared to control group. However, the Russian current did not significantly improve balance performance during eyes-closed velocity tests, suggesting its dependence on visual input to show improvements in balance control. A recent research emphasizes the role of visual feedback and multisensory training in stroke rehabilitation, as these strategies can be very helpful in replacing lost sensory input and improving balance outcome [12]. Our findings are compatible with the increased understanding that RC, if used concurrently with sensory training, might be more effective in addressing the various facets of balance deficit [7].

Our results are consistent with those reported by Joy et al., who found that there was significant improvement in gait speed and lower limb motor recovery after RC addition to conventional exercises [13]. Similarly, Hasan et al. outlined improved results in the 10-meter walk test and Brunnstrom staging after RC application on the quadriceps muscle, confirming the neuromuscular benefits of electrical stimulation in stroke rehabilitation [8].

Wang et al. also observed immediate strength gains in lumbar radiculopathy patients with drop foot after RC application to the anterior tibial muscles. Although this population differs from stroke patients, the mechanism- neuromuscular recruitment and facilitation- supports the present findings [14].

Furthermore, the improvements observed in our study are in agreement with Mayer et al., who showed that RC combined with pharmacologic treatment enhanced gait function and quality of life in individuals with multiple.

sclerosis. This cross-population highlights the broad neuro-modulatory effect of RC in various neurological conditions characterized by motor deficits [15].

Howlett et al. showed the improvement of the toe clearance and the balance of the chronic stroke patients by using of functional electrical stimulation (FES) during walking [16]. Similarly, Aout et al. pointed out that FES contributes to dynamic stability and dorsiflexion improvement, as well as the prevention of the foot drop. These studies support our hypothesis that anterior tibial muscle strengthening is the main factor in the fall risk reduction [17].

The physiological reason for RC’s positive effects is that it can activate a greater number of motor units, including fast-twitch fibers, which are typically difficult to access through normal voluntary contraction in people who have had a stroke [18, 19]. RC makes hypertrophy and neuromuscular re-education possible, thus enabling motor relearning and functional recovery [20]. RC has the ability to fix the problem of arthrogenic inhibition, which is common in post- stroke patients with low volitional control. It increases dorsiflexion in the swing phase of gait, which leads to better static balance and less chance of falling [21]. These results are consistent with the higher BBS scores and the decline in sway indices seen in the experimental group.

The stretching component of our intervention protocol should be mentioned specifically due to its well-known effect in spasticity alleviation, muscle-tendon flexibility improvement, and postural control capacities. Systematic analyses done by Lohmann et al. and Takeuchi et al. have confirmed the significance of chronic stretching interventions, especially when combined with strengthening and proprioceptive training, in achieving sound balance improvement [22, 23]. This integrative approach not only enhances static and dynamic balance but also leads to better functional outcomes, as supported by Hussein et al. [18].

Recent stroke rehabilitation focuses on the importance of multimodal and personalized balance training. Multiple reviews have confirmed that integrating various balance training approaches, such as task-specific, visual feedback, and multisensory interventions, can provide better results than a single modality approach [12]. Robot-assisted balance and core stability exercises were found to be beneficial in dynamic stability and trunk control improvement, while aquatic and dual-task exercises can enhance balance and gait of chronic stroke survivors [24]. Future studies should focus on RC integration with new modalities that can lead to more effective rehabilitation protocols.

## Conclusion

While exercise therapies alone are less efficient, this study demonstrates that RC stimulation targeting the anterior tibial muscle group, combined with a traditional physiotherapy program, improves postural balance and reduces falls in stroke survivors considerably. The Berg Balance Scale and Biodex Balance System showed improvements in both static and dynamic balance metrics. However, the absence of significant improvement under eyes-closed conditions suggests a reliance on visual input and emphasizes the need for more research into multisensory balance training techniques. Overall, RC stimulation presents as a future and beneficial adjunctive intervention in patients with stroke, mainly in improving postural control and dorsiflexion mechanisms. Future studies perhaps will include larger samples and more diverse populations, along with long-term follow-up to monitor the sustainability of these benefits.

### Clinical implications

By integrating RC into the usual rehabilitation protocols, therapists can facilitate postural-control recovery, thus helping the patients to become more independent and less reliant on their caregivers.

### Limitation

The study sample was restricted to patients aged 40–60 years, with a BMI below 30 kg/m², thereby limiting the generalizability of our findings. Lack of objective muscle strength measurements, e.g., dynamometry. The lack of significance in improvements in the eyes-closed tasks further mandates the further investigation of sensory integration and the development of interventions that can address proprioceptive and vestibular deficits. Moreover, the long-term effect is not included in the current study, so further studies should be considered in further research.

## Data Availability

The data presented in the study are available upon request from the corresponding author.

## Notes

**Institutional Review Board Statement**: The study was conducted in accordance with the Declaration of Helsinki and Cairo University Ethical Committee approval of the study protocol (No:116.

## Notes

P.T.REC/012/005504)
